# In Situ Room Temperature Electron-Beam Driven Graphene Growth from Hydrocarbon Contamination in a Transmission Electron Microscope

**DOI:** 10.3390/ma11060896

**Published:** 2018-05-26

**Authors:** Mark H Rummeli, Yumo Pan, Liang Zhao, Jing Gao, Huy Q Ta, Ignacio G. Martinez, Rafael G. Mendes, Thomas Gemming, Lei Fu, Alicja Bachmatiuk, Zhongfan Liu

**Affiliations:** 1 Key Laboratory of Advanced Carbon Materials and Wearable Energy Technologies of Jiangsu Province, Collaborative Innovation Center of Suzhou Nano Science and Technology, Soochow Institute for Energy and Materials InnovationS, College of Physics, Optoelectronics and Energy, Soochow University, Suzhou 215006, China; 1508404030@stu.suda.edu.cn (Y.P.); lzhao@suda.edu.cn (L.Z.); 20114014013@suda.edu.cn (J.G.); huytq8793@gmail.com (H.Q.T.); r.g.mendes@ifw-dresden.de (R.G.M.); alicja-bachmatiuk@wp.pl (A.B.); 2 Centre of Polymer and Carbon Materials, Polish Academy of Sciences, M. Curie-Sklodowskiej 34, Zabrze 41-819, Poland; 3 IFW Dresden, D-01171 Dresden, Germany; i.g.gonzales.martinez@ifw-dresden.de (I.G.M.); t.gemming@ifw-dresden.de (T.G.); 4 College of Chemistry and Molecular Science, Wuhan University, Wuhan 430072, China; leifu@whu.edu.cn; 5 Beijing National Laboratory for Molecular Sciences, Center for Nanochemistry, Beijing Science and Engineering Centre for Nanocarbons, College of Chemistry and Molecular Engineering, Peking University, Beijing 100871, China; zfliu@pku.edu.cn

**Keywords:** graphene, in situ TEM, synthesis

## Abstract

The excitement of graphene (as well as 2D materials in general) has generated numerous procedures for the fabrication of graphene. Here we present a mini-review on a rather less known, but attractive, in situ means to fabricate graphene inside a transmission electron microscope (TEM). This is achieved in a conventional TEM (viz. no sophisticated specimen holders or microscopes are required) and takes advantage of inherent hydrocarbon contamination as a carbon source. Both catalyst free and single atom catalyst approaches are reviewed. An advantage of this technique is that not only can the growth process be imaged in situ, but this can also be achieved with atomic resolution. Moreover, in the future, one can anticipate such approaches enabling the growth of nano-materials with atomic precision.

## 1. Introduction

There are numerous routes with which to obtain graphene which include mechanical exfoliation [1,2,3,4,5,6], chemical exfoliation [7,8,9], reduction of graphene oxide [10,11,12], bottom-up synthesis from molecular precursors [13,14,15], chemical vapor deposition with and without metal catalysts [16,17,18,19], and epitaxial growth over SiC [20,21,22]. In this work however, we review a less well known process for the fabrication of graphene in which hydrocarbon contaminant species in a transmission electron microscope (TEM) are taken advantage of for the in situ fabrication of graphene. In addition, the synthesis reaction is driven solely by the electrons in the imaging electron beam of the TEM. However, before reviewing the in situ synthesis of graphene in a TEM it is useful to first look at the sources of carbon in a TEM and also at electron beam—sample interactions, which drive the reactions for graphene growth.

## 2. Sources of Contamination

**Figure 1:**
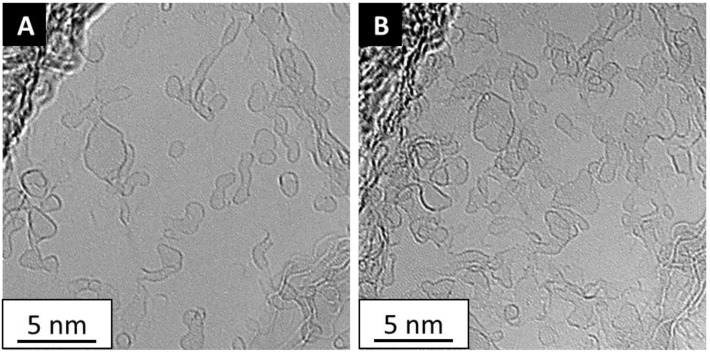
Transmission electron microscope (TEM) micrographs showing the accumulation of column contamination over time. Panel (**A**) Graphene at time = 0, Panel (**B**) Graphene at time = 20 min. The graphene was kept in the TEM column without irradiation for 20 min. The accumulation of contamination on the graphene is obvious. Data collected on an FEI Cs double corrected Titan^3^ at 80 kV.

High vacuum (HV) and ultra-high vacuum (UHV) systems by their nature are designed to, in essence, pump-for-nothing, viz. the goal is to have nothing, or at the very least as few as possible atoms and/or molecules. This in practice is hard to achieve as atoms and molecules tend to adsorb or stick to any surface they come in contact with. As one pumps or evacuates a vacuum chamber adsorbed material can desorb. This desorption can be accelerated by heating the surface. However, once material desorbs it has a very high probability of colliding with a chamber wall again as in a vacuum the mean free path of a gas atom/molecule is large. For example, in UHV (ca. 10^−9^ mbar), a gas molecules mean free path is ca. 40 km! [23]. Thus, in short, HV and UHV chambers tend to have contaminants in the chamber. This process can be seen by placing a relatively clean piece of graphene in a TEM column and leaving it for a period of time under vacuum (without any electron irradiation). After such a step additional deposits can easily be seen on the graphene, as for example shown in Figure 1. In a TEM, a vacuum chamber is necessary not only to allow the electrons to traverse through the microscope’s optical system without scattering, but also to minimize contamination that often leads to unwanted growth of carbonaceous material over a sample as this reduces contrast and thus diminishes the observable detail. Within the TEM column (where the sample resides) the vacuum pressure is ca. 10^−7^ mbar (HV) and thus while contaminant molecules in the chamber are reduced they are not removed. Moreover, in addition to contamination from the TEM vacuum chamber (typically referred to as the column), the sample itself can introduce additional contamination and moreover, even if not, the sample itself can adsorb contaminants prior to insertion in the TEM. Specimen based contamination is considered the main contributor, in particular, for hydrocarbon contamination [24,25]. The process of changing the sample also introduces the sample holder itself to contamination. In short, there are various sources of contaminant molecules and these include hydrocarbons, air, and water vapor. Hydrocarbon contamination can be particularly problematic because when adsorbed on a sample and irradiated with electrons, cross-linking of these adsorbed organic molecules leads to the formation of a carbonaceous film on the sample surface [26,27,28,29,30,31,32]. This process is usually referred to as electron beam induced deposition (EBID). While this is often an unwanted process in a TEM, as we shall see further on, at times, this can be useful. (Note: as a side note, there are strategies to reduce contamination, including extended evacuation of the sample holder in the entry lock prior to insertion to the TEM column as well as extended evacuation in the column which can also be carefully baked out to further reduce contamination). 

## 3. Electron–Sample/Contamination Interactions

In terms of the electron sample interactions (scattering) at the sample, they can be classified into elastic and inelastic events. When an electron from the TEM electron beam undergoes an electrostatic deflection by the Coulomb field of an atom in the sample, this is referred to as elastic scattering. In the case of inelastic scattering, a Coulomb interaction of incoming electrons with electrons surrounding the atoms forming the specimen takes place. These processes are of course important for imaging and diffraction in electron microscopes [33]. They can also lead to damage or modification processes. Simplified, we can place the damage/modification processes into three categories; Radiolysis, heating, and knock-on damage, also often referred to as sputtering. Radiolysis refers to electron–electron interactions which can break chemical bonds and is an inelastic scattering process. This can lead to atom loss, and hence mass loss. Radiolysis is dependent on the irradiating electron dose (C/m^2^) and the process becomes more relevant as the irradiating electron energy (E_o_) decreases. It also depends on the bond type of the specimen or contaminant. Heating which also arises due to inelastic scattering occurs because electrons have similar mass and thus significant energy can be transferred leading to a local temperature rise. Heating increases with increasing electron current density and varies with the material’s thermal conductivity. Knock-on damage is an elastic scattering event between the irradiating electrons and the sample or contaminant atom nucleus. Since the event is elastic there is no change in the irradiating electrons energy, however, energy is transferred from the irradiating electrons since the electrons are deflected by the atom nucleus and so, to conserve energy and momentum, some energy must be transferred. This is akin to bremsstrahlung radiation, except here, the energy release is not in the form of an X-ray, rather energy transfers as momentum to the atom and can be sufficient to remove the atom itself, hence the term sputtering. The process depends on the incoming electron energy and knock-on damage threshold of the material.

## 4. In Situ Graphene Growth

Of the published data there are two primary approaches to take advantage of (contaminant) hydrocarbon species inside a TEM column as a carbon feedstock for graphene synthesis driven by the electron beam. In the first process no catalyst is required, but can be considered a two-step process where initially carbonaceous material is formed through electron beam induced deposition, and then graphitized with further electron beam irradiation (typically under different conditions). In the second process, a catalyst (a single metal atom) is used to presumably aid cracking (decomposition) of the hydrocarbon and growth of graphene. We first review the case of catalyst free grown graphene and then catalyst assisted growth of graphene. It is also worth pointing out at this stage that all the experiments reviewed here were conducted at room temperature, and calculations show that in all cases the temperature increase due to electron beam irradiation is negligible.

### 4.1. Catalyst Free In Situ Graphene Growth

**Figure 2:**
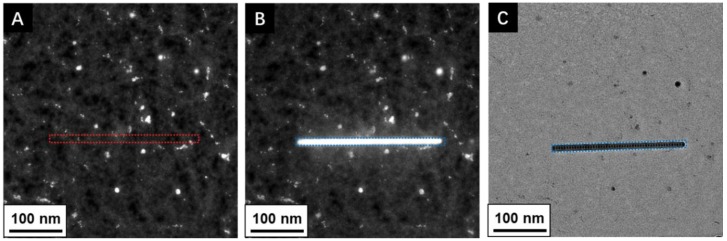
S/TEM images showing amorphous carbon deposited on graphene using electron beam induced deposition (EBID) in the scanning probe mode (STEM). (**A**) STEM image of graphene substrate. The red box indicates the line scanning position to be deposited (STEM mode). (**B**,**C**) the blue box shows formation amorphous carbon at the scanned line position (high contrast); B STEM image and C TEM image. Data collected on a FEI Titan Themis^3^ with Cs correction for the objective lens at 80 kV.

**Figure 3:**
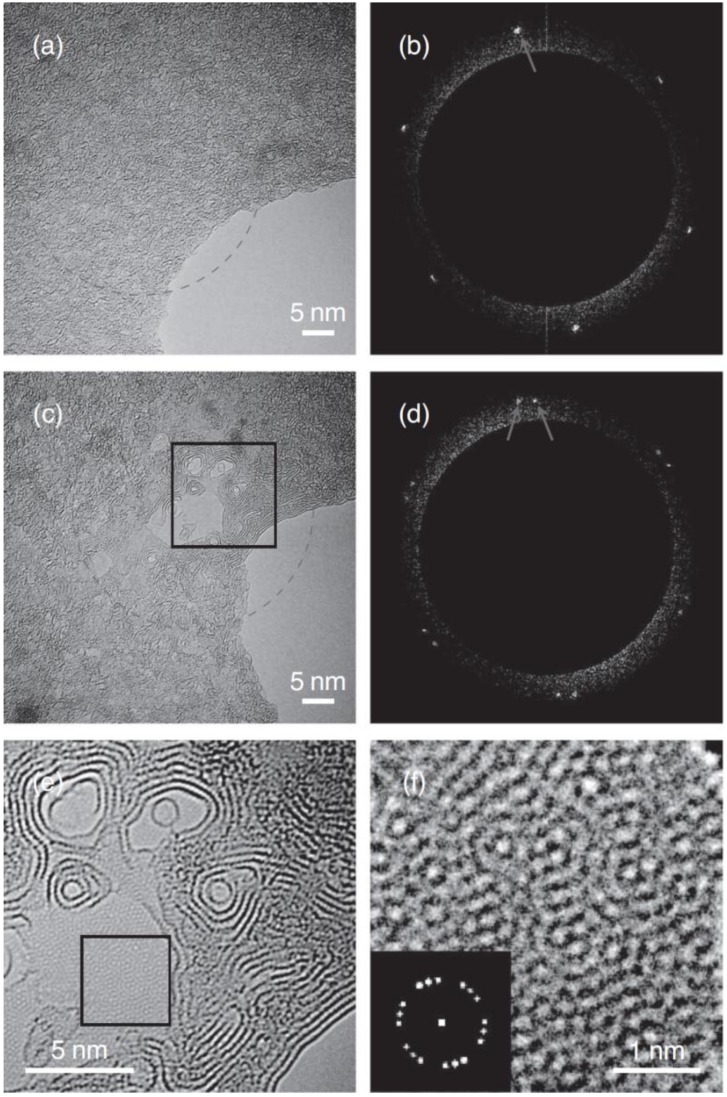
Catalyst-free fabrication of graphene from amorphous carbon supported on graphene. (**a**) Pristine sample of amorphous carbon residing on a single graphene layer. The dashed ring indicates the area to be irradiated. (**b**) The Fourier transform from micrograph shown in panel (a)—six spots from the underlying single layer graphene support are visible. (**c**) The sample shown in panel (a) after 12 min irradiation. (**d**) The Fourier transform from micrograph (c)—An additional set of spots as compared to before irradiation (see panel (b)) have now appeared confirming new graphene has formed on the graphene support. (**e**) Magnified section from (b) showing terrace steps of the grown planar few-layer graphene. (**f**) Further magnified section from (e) showing Moiré patterns of the newly formed few-layer graphene. Inset: corresponding Fourier transform indicating three different rotations from rotational sticking faults between the graphene layers. Reproduced with permission. Ref. [34] Copyright (2012), John Wiley and Sons.

As mentioned above in this graphene fabrication process, two steps are required [34]. In the first step carbonaceous material is deposited (in this case one can consider this as the deposition of amorphous carbon). The amorphous carbon deposition, usually referred to as EBID, is argued to occur through complex electron beam interactions (radiolysis) of adsorbed hydrocarbons on the surface of the substrate [35]. In this case the substrate is graphene. EBID is generally accomplished in the scanning probe mode (STEM) as the current densities are much higher (as compared to TEM (parallel beam), and even in STEM mode, the process is sensitive to the beam focus [35]. Figure 2 shows EBID deposits on graphene. In a work Boerrnert et al. [34], amorphous carbon is deposited on chemical vapor deposition (CVD) grown graphene using EBID in the STEM mode at 80 kV (so as not to damage the underlying graphene). After this the TEM is then switched to the TEM mode and then the amorphous carbon is graphitized by extended exposure to the electron beam (at 80 kV). It is well known that electron beam irradiation can graphitize amorphous carbon through radiolysis reactions (breaking bonds and allowing diffusion and rearrangement of C species) [36,37]. If freestanding amorphous carbon is irradiated, sp^2^ carbon onions form (spherical graphene cages within each other) [38,39]. The process of forming a graphitic spherical structure is argued to occur so as to avoid dangling bonds and to distribute strain evenly and in this way seek energy minimization [38]. However, in the work of Boerrnert et al., where the amorphous carbon was supported by a graphene or hexagonal boron nitride (hBN) substrate, something rather different was observed. In this case, the amorphous carbon formed few-layer graphene parallel to the support surface (see Figure 3). To understand why this occurs (as opposed to carbon onion formation) one can look at a study by Barreiro et al. [40] in which amorphous carbon over graphene is graphitized by current annealing. Supporting molecular dynamics studies showed the graphitization led to graphene layers forming parallel to the substrate due to the van der Waals interactions between the amorphous carbon and underlying support. In the same way, this explains the formation of the parallel graphene formed in situ in a TEM by the electron beam when the initial amorphous carbon resides on a support. Electron induced heating calculations for this study confirmed temperature increases (due to the electron beam) are negligible, in agreement with studies for carbon onion formation [41,42,43].

In summary, in this two-step process, amorphous carbon is first deposited by EBID in the STEM mode, and then graphitized through radiolysis reactions by the electron beam in the TEM mode.

### 4.2. Catalyst Assisted In Situ Graphene Growth

**Figure 4:**
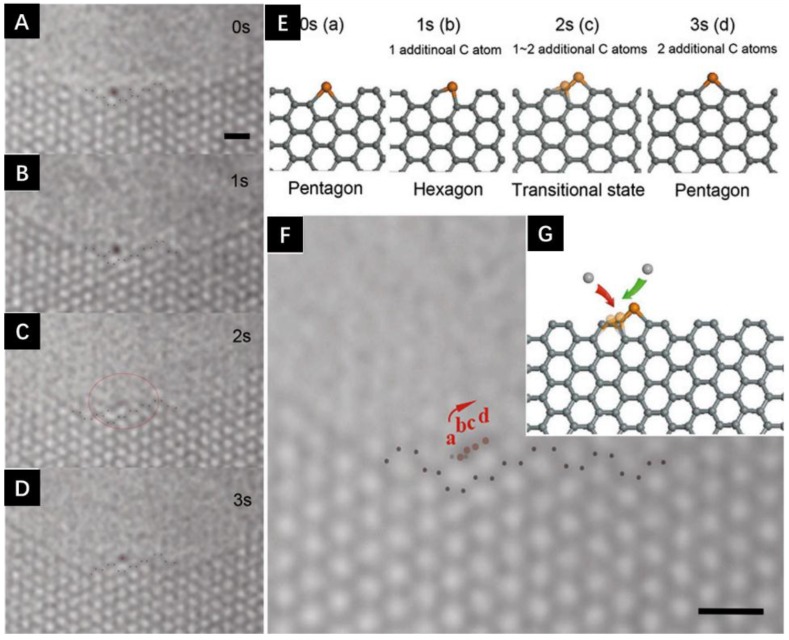
Catalytic growth of graphene edge by single Fe atom. (**A**–**D**) show the motion of the Fe atom along the pore edge. (**E**) The atomic structures for A–D. (**F**) The combination of A–D, which shows the trace of the Fe atom during the one-unit cell translocation. (**G**) The atomic structure for the whole growth process. All scale bar: 0.5 nm. Reproduced with permission. Ref. [45] Copyright (2014), National Academy of Sciences.

In the case of catalyst assisted growth of graphene from hydrocarbon contaminants inside a TEM, thus far, all reported cases were conducted at 80 kV in the high resolution TEM (HRTEM) mode. Chronologically, the first reported case was that in which a single Fe atom at a graphene edge would diffuse or move along the edge while under electron irradiation [44]. The diffusion was anomalous (non-Brownian motion was observed) and most interestingly, as the Fe atom moved, new C atoms were removed from the graphene edge (etching) or added to the carbon edge, viz. graphene growth. Figure 4 shows an example of graphene growth from a single Fe atom [45]. Supporting high temperature density functional tight binding molecular dynamics (DFTB-MD) calculations where implemented to emulate the catalytic process. By conducting the simulations at high temperature, the process is essentially accelerated thus making the simulation feasible. The theoretical studies confirmed the catalytic role of Fe atoms for the growth of graphene. Density functional theory (DFT) allowed the C atom (addition) structural configurations to be determined during the graphene growth and they fitted well with the experimental observations. Fe atoms were shown to bond at the edge (C atoms) in six configurations. The two most relevant, though, are when an Fe atom substitutes two C atoms forming a pentagon ring or a single Fe atom substitutes a single C atom thus forming a hexagon. The pentagonal structure and change to a hexagonal structure by incorporating a C atom (growth). A hexagonal structure can lose the Fe atom and replace it with a C atom (growth). In this way a Fe atom can diffuse along the edge and add carbon atoms, viz. growth. From this (and other studies) it remains unclear if the metal catalyst atom also helps decompose hydrocarbons in the system or if this is achieved solely by the electron beam.

**Figure 5:**
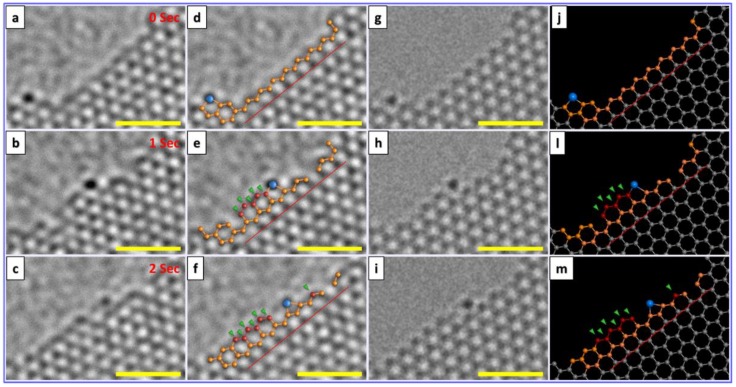
Electron beam driven catalytic growth of graphene by a single Cr atom at the graphene edge. (**a**–**c**) HRTEM images showing in-situ growth process; (**d**–**f**) with partial stick and ball models to assistance viewing; (**g**–**i**) image simulations showing the growth process; (**j**–**m**) complete stick and ball models. Blue balls are designated to Cr while red balls and green arrows signify new C atom. All scale bars are 1 nm. Reproduced with permission from Ref. [46] Copyright (2017), Tsinghua University Press and Springer-Verlag GmbH Germany.

**Figure 6:**
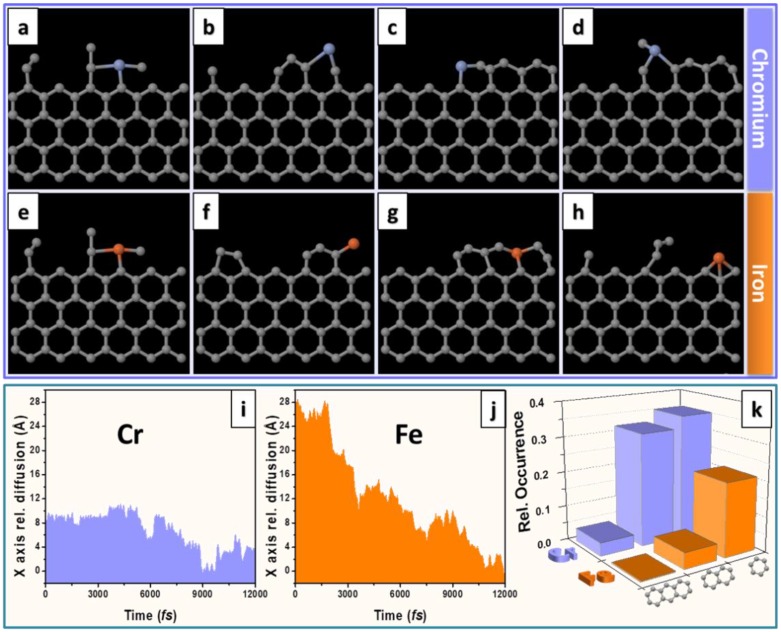
Stick and ball models from molecular dynamics (MD) simulations for a Cr atom (**a**–**d**) and Fe atom (**e**–**h**) at a graphene edge showing catalytic growth of graphene. Comparison of the diffusion activity of Cr and Fe atoms at graphene edge (**i**,**j**) and relative occurrence of new hexagon formation at the graphene edge (**k**). Reproduced with permission from Ref. [46] Copyright (2017), Tsinghua University Press and Springer-Verlag GmbH Germany.

A more recent study by Ta et al, explored Cr atoms at the open edge of graphene layers while under electron beam irradiation (80 kV) [46]. Again the electron beam energy employed was 80 kV. In their study a number of different Cr atoms at graphene edges where investigated. In all cases they observed only growth i.e. no etching processes were seen. Figure 5 shows the growth of graphene from a Cr atom. DFT studies to investigate the energetic and kinetic stability of Cr and Fe suggests a slightly higher binding energy for Cr as compared to Fe. Complimentary MD studies showed that the diffusion of Cr atoms and Fe atoms at carbon edges were different in that Cr atoms diffuse at a lesser rate than Fe, in other words, they are more stable. This suggests Cr is a more effective metal atom catalyst than Fe since an efficient catalyst requires a good balance between stability and migration. Stability improves the efficacy of C attraction for growth, and migration allows for C incorporation. The MD studies showed greater hexagon formation per unit time for Cr as opposed to Fe (Figure 6) again supporting the concept that single Cr atoms are better than single Fe atoms for graphene growth under these conditions. The MD simulations were performed based on the Born–Oppenheimer approximation (BOMD) and as for the case for the Fe single atom catalytic growth of graphene, the MD were conducted at high temperature to allow the simulation to occur in a reasonable time. Following In a similar study, the same group showed how a Cr catalyst atom could heal a hole in graphene. In this case the carbon was not supplied from a hydrocarbon contaminant, but rather from a contaminating carbon nanotube. The process also transformed the carbon nanotube in to a fullerene [47].

Such studies are important to better comprehend single atom growth of graphene (and 2D materials in general) and could lead to precision growth of nano-materials at the atomic scale.

## 5. Summary and Future Thoughts

In this mini review we presented two primary routes in which contaminant molecules, in particular hydrocarbons, present inside a TEM can be used as a carbon source for in situ graphene synthesis driven by electron beam irradiation. Two primary approaches are presented. In the first, a two-step procedure is followed in which hydrocarbon contaminants are decomposed by the electron beam (in STEM mode) to form an amorphous carbon film (so called EBID) over a support. In the second step, the amorphous carbon is transformed to graphene layers parallel to the support surface by an electron beam in the TEM mode. This graphene synthesis procedure does not require a catalyst, while the second approach does, and implements a single metal atom catalyst for the graphitization process. In effect, a single metal atom (Fe or Cr) at an open edge graphene is able incorporate C atoms (growth) as it diffuses along the graphene edge.

These studies are important in that they help us understand the growth of graphene at the atomic scale and also pave the way for techniques to study single atom catalysis. Such work can lead to atomically precise growth of 2D materials which could have important implications for the microelectronics industry. Moreover, it is conceivable that such work could be used for lateral hetero 2D materials, for example, stitching graphene into hBN.
